# Role of E3 ubiquitin ligases and deubiquitinating enzymes in SARS-CoV-2 infection

**DOI:** 10.3389/fcimb.2023.1217383

**Published:** 2023-06-09

**Authors:** Mingjiu Zhao, Mengdi Zhang, Zhou Yang, Zhiguang Zhou, Jiaqi Huang, Bin Zhao

**Affiliations:** ^1^ National Clinical Research Center for Metabolic Diseases, Metabolic Syndrome Research Center, Key Laboratory of Diabetes Immunology, Ministry of Education, and Department of Metabolism and Endocrinology, The Second Xiangya Hospital of Central South University, Changsha, Hunan, China; ^2^ Xiangya School of Public Health, Central South University, Changsha, China; ^3^ Furong Laboratory, Central South University, Changsha, China

**Keywords:** ubiquitin, E3 ubiquitin ligases, deubiquitinating enzymes (DUBs), SARS-CoV-2, COVID-19

## Abstract

Ever since its emergence in 2019, COVID-19 has rapidly disseminated worldwide, engendering a pervasive pandemic that has profoundly impacted healthcare systems and the socio-economic milieu. A plethora of studies has been conducted targeting its pathogenic virus, SARS-CoV-2, to find ways to combat COVID-19. The ubiquitin-proteasome system (UPS) is widely recognized as a crucial mechanism that regulates human biological activities by maintaining protein homeostasis. Within the UPS, the ubiquitination and deubiquitination, two reversible modifications, of substrate proteins have been extensively studied and implicated in the pathogenesis of SARS-CoV-2. The regulation of E3 ubiquitin ligases and DUBs(Deubiquitinating enzymes), which are key enzymes involved in the two modification processes, determines the fate of substrate proteins. Proteins associated with the pathogenesis of SARS-CoV-2 may be retained, degraded, or even activated, thus affecting the ultimate outcome of the confrontation between SARS-CoV-2 and the host. In other words, the clash between SARS-CoV-2 and the host can be viewed as a battle for dominance over E3 ubiquitin ligases and DUBs, from the standpoint of ubiquitin modification regulation. This review primarily aims to clarify the mechanisms by which the virus utilizes host E3 ubiquitin ligases and DUBs, along with its own viral proteins that have similar enzyme activities, to facilitate invasion, replication, escape, and inflammation. We believe that gaining a better understanding of the role of E3 ubiquitin ligases and DUBs in COVID-19 can offer novel and valuable insights for developing antiviral therapies.

## 1 Introduction

UPS is a complex cellular process that plays a crucial role in maintaining the balance of proteins within cells, regulating various cellular processes such as the progression of cell cycle, DNA repair, and transcription (Reed, 2006; Daulny and Tansey, 2009; Storici, 2011). UPS entails two opposing processes that perform a crucial role in sustaining protein homeostasis. One process involves the conjugation of a small protein, ubiquitin, to a substrate, which allows for the degradation of the target protein by the subsequent proteasomal pathway. This process comprises a triad of enzymes, namely E1 (ubiquitin-activating enzyme), E2 (ubiquitin-conjugating enzyme), and E3 (ubiquitin ligase) (Daulny and Tansey, 2009). The other process is referred to as deubiquitination, which is the reversal of ubiquitination. DUBs are the key enzymes that execute this process by detaching the ubiquitin tag from the substrate protein, thereby preventing undue degradation of the protein (Amerik and Hochstrasser, 2004). The vital role of ubiquitination and deubiquitination in regulating cellular activities is apparent from the functional changes observed in key enzymes, including E3 enzymes and DUBs, in diseases such as cancer, autoimmune disorders, and infectious diseases, including COVID-19 (Isaacson and Ploegh, 2009; Sun et al., 2020; Yadav et al., 2022).

Since COVID-19 broke out in 2019, the number of confirmed global cases has surpassed 700 million as of April 6, 2023 (WHO COVID-19 Dashboard, 2020). The pandemic’s devastating impact is partly attributed to the extraordinary replication and transmission ability of the pathogenic SARS-CoV-2 virus, coupled with its ability to evade immune surveillance in the human body. Although a considerable portion of those infected with COVID-19 may only manifest mild or no symptoms at all (Vetter et al., 2020), the disease has nonetheless claimed the lives of millions (WHO COVID-19 Dashboard, 2020). Thus, it is necessary to have an evolving understanding of the pathogenic mechanisms of SARS-CoV-2 to develop effective prevention and treatment strategies against the ongoing COVID-19 pandemic.

Transmission of COVID-19 is largely contingent upon the roles played by its viral proteins, comprising 4 structural proteins, 16 non-structural proteins (nsps), and a series of accessory proteins (Yang and Rao, 2021). The structural proteins of SARS-CoV-2 include the spike (S) protein, the envelope (E) protein, the nucleocapsid (N) protein, and the membrane (M) protein. The S protein is a homotrimer anchored to the virus surface that mediates SARS-CoV-2 attachment to host cell membranes and subsequent fusion through binding to host receptors such as angiotensin-converting enzyme-2 (ACE2) (Belouzard et al., 2012). The E protein primarily facilitates the assembly, budding, and release of the virus, and also participates in inflammasome activation (Schoeman and Fielding, 2019). As the only structural protein located inside the viral particle, the N protein packages the viral RNA genome, helps form the ribonucleoprotein complex, provides protection to the viral RNA, and participates in virus replication and transcription (McBride et al., 2014). The M protein can interact with N protein and E protein to promote virus assembly and release (Corse and Machamer, 2003; Yuan et al., 2021; Zhang et al., 2022). Apart from these viral proteins, two proteases involved in the generation of nsps, namely the main protease (Mpro) and the papain-like protease (PLpro), play indispensable roles in the transmission and pathogenesis of the virus.

SARS-CoV-2 infection leads to changes in the ubiquitination system, affecting E3 ligases, DUBs, and enzymes involved in modifying and removing ubiquitin-like molecules (Gao et al., 2021; Selvaraj et al., 2021; Vanderboom et al., 2021; Baskol et al., 2022). Furthermore, the observation of the interaction between key enzymes of the ubiquitin system and viral proteins provides strong evidence for the pivotal role of ubiquitin-mediated regulation in the activity of SARS-CoV-2 (Somasekharan and Gleave, 2021; Stukalov et al., 2021; Bing et al., 2022). Thus, this review expounds upon the host’s employment of E3 ligases and deubiquitinases (DUBs), the key enzymes mediating ubiquitination and deubiquitination, to counteract viral invasion and pathogenesis, and how the SARS-CoV-2 utilizes its viral proteins to compete host control mechanisms, evade immune surveillance, and facilitate viral replication and transmission. Additionally, this review expounds on the roles of other ubiquitin-like modifications, such as ISGylation and SUMOylation, in disease regulation.

## 2 SARS-CoV-2 virus invasion

The invasion of the virus occurs through the binding of its protein structure to host receptors, and the SARS-CoV-2 Spike (S) protein specifically targets ACE2 as a crucial binding site. However, recent studies have indicated that the ubiquitin E3 ligase may interfere with this interaction process. Transmembrane serine protease type 2 (TMPRSS2) is essential for the virus to bind to ACE2 and enter host cells (Hoffmann et al., 2020), and DDB1- and CUL4-associated factor 1 (DCAF1), an E3 ubiquitin ligase component, can ubiquitinate and degrade TMPRSS2, hindering the binding of the S protein to ACE2 (Chen et al., 2021).

In addition, E3 ubiquitin ligase can directly regulate the levels of ACE2. Wang et al. found that hospitalized smokers had a lower incidence of COVID-19 during the pandemic. Further investigation showed that cigarette smoke extract (CSE) and carcinogen benzo(a)pyrene (BaP) activate the catabolism of the ACE2 protein through the ubiquitin E3 ligase Skp2 (S-phase kinase-associated protein 2). This, combined with the interference of tobacco carcinogens on the function of the SARS-CoV-2 S protein, renders smokers less susceptible to SARS-CoV-2 infection (Wang et al., 2021). In line with this finding, another study demonstrated that cell cycle arresting compounds could decrease Skp2 expression and lead to a reduction in ACE2 degradation, which promotes the entry of SARS-CoV-2 into host cells (Xiao et al., 2023). Targeting these E3 ligases is one mechanism of virus invasion. For instance, the S protein of the virus can promote the dissociation of ACE2 from the E3 ligase UBR box N-recognin-4 (UBR4) (Chuang et al., 2022), thereby stabilizing the levels of ACE2. Deubiquitination by ubiquitin carboxyl terminal hydrolase 1 (UCHL1) can also contribute to the stabilization of ACE2 (Bednash et al., 2023). Unlike the process of ubiquitination, which marks proteins for degradation, the action of the E3 small ubiquitin-like modifier (SUMO) ligase protein inhibitor of activated STAT 4 (PIAS4) in ACE2 SUMOylation can actually stabilize the ACE2 protein and further promote SARS-CoV-2 infection (Jin et al., 2022).

## 3 SARS-CoV-2 virus replication, assembly, and release

The SARS-CoV-2 RNA polymerase serves as the fundamental structure for the virus’ transcription and replication, with nsp12 being a crucial component of the polymerase (Wang et al., 2020). An investigation has revealed that elevated temperatures can impede the virus by facilitating nsp12 ubiquitination mediated by E3 ubiquitin ligase Zinc Finger Protein 598 (ZNF598), leading to a reduction in the quantity of viral RNA copies and a decrease in the viral concentration (Maimaitiyiming et al., 2022). Additionally, it is widely recognized that Mpro, a kind of 3CLpro, plays a crucial role in the viral replication process due to its ability to cleave viral polyproteins, releasing nsps that initiate subsequent virus transcription. However, the role of Mpro in viral replication through the cleavage of host proteins related to the ubiquitination pathway is also noteworthy (Prescott, 2022). Another study identified E3 ligase ring finger protein 20 (RNF20) as a target protein of 3CLpro and demonstrated that impairment of RNF20 interferes with its regulation of the ubiquitination of sterol regulatory element binding protein 1 (SREBP1), thereby promoting virus replication (Zhang et al., 2021).

Unlike RNF20, RNF5 has been found to be involved in regulating viral assembly and budding. RNF5 exerts an inhibitory regulatory effect on virus assembly and budding through ubiquitination of the E protein (Li et al., 2023). However, in contrast to this discovery, another study showed a beneficial influence by RNF5 promoting the interaction between the M protein and E protein. This study also mentioned Pad-One-Homologue1 (POH1), a deubiquitinase negatively regulates this ubiquitination process (Yuan et al., 2021). The contradictory reports on the role of RNF5 in regulating virus assembly and release suggest a more complex role for RNF5 in this regulatory process than previously thought, which may require further research in the future to fully understand.

## 4 Host antiviral response and SARS-CoV-2 immune evasion

The efficient degradation of viral invasion-related S protein is vital for an effective antiviral response in the host. Recent research suggests that the E3 ubiquitin ligase MARCH8 may regulate this process, as it has been found to inhibit SARS-CoV-2 S protein in a dose-dependent manner (Yanzhao et al., 2022). Its mechanism may be linked to the retention of S protein in an internal, LAMP-1+ compartment (Lun et al., 2021). Another E3 enzyme, tripartite motif-containing protein 21 (TRIM21), also targets the S protein to inhibit viral infection ability (Chatterjee et al., 2020). Besides the S protein, some nsps have also been identified as targets for E3 enzymes in the fight against viral infection. The E3 ubiquitin ligase TRIM7 degrades nsp5 and nsp8 by binding to the glutamine-end motif, which partially restores the interferon response that is inhibited by these proteins (Liang et al., 2022). Moreover, the ubiquitination of host proteins targeted by the virus is another approach for combating viral invasion. Specifically, site-specific ubiquitination on Rab7, a GTPase, can weaken the virus’s ability to invade host cells by inhibiting its colocalization with ORF3a of SARS-CoV-2 (Jung et al., 2022).

The human body can defend itself against viral invasion by degrading viral proteins through ubiquitination. However, SARS-CoV-2 can hijack the host’s deubiquitinating enzyme system to counteract the antiviral response. For example, as a protein that interacts with SARS-CoV-2, ubiquitin-specific peptidase 13 (USP13) displays a considerably high incidence of genomic alterations within immune cells of SARS-CoV-2 patients (Süt, 2020). Although USP13 assumes an antiviral role during the pathogenesis of SARS-CoV-2 (Ravindran et al., 2022), some contend that in specific scenarios, USP13 not only fails to impart antiviral effects but instead stimulates viral invasion. This is due to their discovery that SARS-CoV-2 hijacks the host’s USP13 to impede nsp13 degradation through ubiquitination, resulting in obstruction of antiviral constituents’ functionality and inhibition of type I IFN production (Guo et al., 2021). Moreover, other USP family members, USP25 and USP29, also contribute significantly to the pathogenicity of SARS-CoV-2 (Gao et al., 2022; Kim et al., 2023). The contribution of USP25 arises from its protective deubiquitinating effect on nsp16 and its associated complex which aids the virus RNA in evading immune system surveillance through methylation modification (Alshiraihi et al., 2021). And ORF9b, which is protected by USP29, plays a vital role in countering the host’s interferon response to SARS-CoV-2 (Han et al., 2021).

Viruses can also use ubiquitination modification to enhance their virulence. Israeli, M. and colleagues identified the CUL5 gene as a potential facilitator of viral infection. This gene codes for the Cullin5 protein, which is a crucial component of several E3 ubiquitin-protein ligase complexes. Their findings suggest that the SARS-CoV-2 virus may have adapted E3 enzyme-mediated ubiquitination modification and utilized it to promote its pathogenicity under specific circumstances (Israeli et al., 2022). Similarly, two studies on the ORF7a viral protein indicate that the SARS-CoV-2 virus has a potent ability to exploit the host’s ubiquitin system. By utilizing the host’s ubiquitin system to ubiquitinate ORF7a, SARS-CoV-2 disrupts IFN responses and helps infected cells evade apoptosis, thus facilitating viral replication (Cao et al., 2021; Liu et al., 2022).

The IFN response is a significant element of the innate immune system, and its indispensable role in the anti-SARS-CoV-2 response is indisputable. The activation of IFN production is controlled by multiple steps involving E3 enzymes and DUBs regulation. For instance, the interferon-stimulated gene (ISG) XAF1, can initiate IFN-triggered antiviral immunity, possibly by counteracting the CHIP-mediated ubiquitination and degradation of IFN regulatory factor 1 (IRF1), a nuclear factor that stimulates the IFN-I gene promoter (Han et al., 2022). Moreover, Pattern recognition receptors (PRRs) can detect viruses and trigger pathways to induce IFN production. RNA helicase DHX16, a potential PRR, can amplify the activation of the IFN-I production pathway. The process requires DHX16 to interact with unanchored K48-poly-Ub mediated by E3-Ub ligase TRIM6 (Hage et al., 2022). And for another recently discovered PRR, stimulator of interferon genes protein (STING), which has been shown to have anti-SARS-CoV-2 effects, and the deubiquitinase USP22 may regulate the IFN signaling through ubiquitination modification that involves STING’s physical scaffold (Li et al., 2021; Karlowitz et al., 2022).

To conflict host’s IFN response, SARS-CoV-2 specifically targets various stages of the IFN response to evade host immunity through the ubiquitination of key proteins. For instance, the ubiquitination of STING is impeded by the 3Clpro of SARS-CoV-2, which leads to its impaired functionality (Rui et al., 2021). Moreover, SARS-CoV-2 uses E3 ligases, such as host RNA-binding antiviral protein TRIM25 (Lee et al., 2021), to regulate retinoic acid-inducible gene I (RIG-I), another PRR, for counteracting host immune responses. On one hand, SARS-CoV-2 can interfere with TRIM25 E3 ligase-mediated ubiquitination of RIG-I activity (Wu et al., 2020), while on the other hand, it can promote TRIM25 and STIP1 homology and U-Box containing protein 1 (STUB1)-mediated ubiquitination of RIG-I degradation to ultimately interfere with IFN production (Zhang et al., 2021; Zhao et al., 2021). Activated RLRs can transmit signals to downstream mitochondrial antiviral signaling (MAVS), which further activates various kinases such as TANK-binding kinase 1 (TBK1) and NF-κB essential modulator (NEMO) for subsequent IFN signaling (Seth et al., 2005) ^(p3),^ (Rothwarf et al., 1998). However, MAVS, TBK1, and NEMO have been identified as targets for SARS-CoV-2 to evade innate immunity through ubiquitination. Nsp5 can act as an E3 ligase to promote ubiquitination degradation of MAVS (Liu et al., 2021), while the N protein inhibits TRIM31-mediated MAVS activation of multiubiquitination and aggregation (Wang et al., 2021). For the kinases, the virus M protein promotes TBK1 ubiquitination degradation (Sui et al., 2021), while ORF9b inhibits NEMO activation of multiubiquitination (Wu et al., 2021). Ubiquitination not only participates in SARS-CoV-2 interference with IFN production but also impedes IFN function. Ubiquitination degradation of the interferon receptor subunit 1 (IFNAR1) leads to SARS-CoV-2-infected cells developing tolerance to IFN (Chen et al., 2021). And SARS-CoV-2 disrupts the antiviral effects of interferon (IFN) by upregulating suppressor of cytokine signaling 1 (SOCS1), which recruits the E3 ubiquitin ligase adaptors elongins-B/C to speed up the ubiquitin degradation of Janus kinase 2 (JAK2), an important kinase in downstream IFN signaling (Rong et al., 2021).

## 5 SARS-CoV-2-induced host damage

### 5.1 Excessive inflammation and cytokine storm

While the innate immune response is crucial in defending against SARS-CoV-2, excessive immune activation can cause severe cytokine storms, even leading to worsening and potential multiple organ failure (Song et al., 2020). Researchers have found that during SARS-CoV-2 infection, E3 ligase cellular inhibitor of apoptosis (cIAP) and linear ubiquitin chain assembly complex (LUBAC) can mediate the ubiquitination of Z-DNA-binding protein 1 (ZBP1), a PRR, and its scaffold RIP kinases at K63 and M1, which promotes ZBP1 signal transduction and leads to cytokine production. While ZBP1-induced ubiquitin-dependent NF-κB signaling can have antiviral effects, the authors also noted that ZBP1 may contribute to pathogenic type-I IFN responses (Peng et al., 2022). Moreover, studies have shown that viral proteins nsp6 and ORF7a, with the participation of E3 enzymes TRIM13 and RNF121, can directly recruit transforming growth factor-β-activated kinase 1 (TAK1) and IκB kinase (IKK) complexes in a ubiquitin-dependent manner to activate NF-κB signaling and promote the production of numerous pro-inflammatory factors (Nishitsuji et al., 2022). Some antiviral ubiquitin-like modifications (Perng and Lenschow, 2018) have also been demonstrated to regulate SARS-CoV-2-induced cytokine storm. For instance, viral nsp5 enhances the NF-κB signal by stabilizing MAVS through SUMOylation (Li et al., 2021). Blocking neddylation can prevent the activation of PBMCs, ultimately leading to a decrease in cytokine production (Serrano-Maciá et al., 2022). And symptomatic COVID-19 patients have been shown to have higher ISGylation levels in peripheral blood mononuclear cells (PBMC) (Schwartzenburg et al., 2022). However, Cao X believes that extracellular free ISG15 caused by SARS-CoV-2 deubiquitination of ISGylation is the main cause of SARS-CoV-2-induced inflammation (Cao, 2021). The virus-induced excessive inflammation can lead to damage in various tissues and organs, such as the nerves and kidneys. Viral microRNA can disrupt the stability of IRF9 by inhibiting USP33, which hinders its role in regulating inflammation, ultimately causing activation of human microglia and nerve injury (Mishra and Banerjea, 2021). The viral protein ORF3A utilizes the E3 enzyme TRIM59 to modulate the activation of the STAT3 signal, thereby causing SARS-CoV-2-related renal tubular cell injury (Cai et al., 2023).

### 5.2 Complications and comorbidities

A study has shown that protein ubiquitination exacerbates disease severity in affected individuals, which may imply its involvement in the development of complications (Huang et al., 2022). Research posits that the deubiquitinase UCHL1 potentially participates in the neuropathological damage and ensuing complications that stem from SARS-CoV-2 infection and may also possess prognostic value (De Lorenzo et al., 2021; Liu et al., 2021; Tokic et al., 2022). The M protein inhibits B-cell lymphoma 2 ovarian killer (BOK) by ubiquitinating it. This results in apoptosis of lung cells, increased lung permeability, and ultimately pulmonary edema. The injury can progress to a severe complication-respiratory failure (Yang et al., 2022). In addition to severe complications, some patients experience loss of smell and taste (Bagheri et al., 2020; Makaronidis et al., 2020), potentially due to SARS-CoV-2 ORF10’s promotion of the ubiquitination of cilia function-associated proteins by the E3 ligase CRL2(CUL2^ZYG11B^)complex, leading to cilia dysfunction with odorant receptor expression (Wang et al., 2022).

Additionally, SARS-CoV-2 can modulate the progression of other concurrent diseases by manipulating the ubiquitination pathway. By co-opting the host ubiquitination system, SARS-CoV-2 can transform host proteins into autoantigens and aggravate autoimmune diseases (Wang et al., 2021). The virus may also impact cancer by involving E3 ligases in its action. SARS-CoV-2 has been found to leverage E3 ligases to modulate the RAS family and exert influence on the tumor microenvironment, thereby potentially affecting tumor development (Cui et al., 2021). Meanwhile, there appears to be a mutual interaction between SARS-CoV-2 and tumors, as tumor patients exhibit increased susceptibility to SARS-CoV-2 infection, and Temena et al. propose that the E3 linking enzyme TRIM31 may facilitate this process (Temena and Acar, 2022).

## 6 DUB and de-ISGylating activity of PLpro

In addition to manipulating host’s ubiquitin regulation system, SARS-CoV-2 can also encode PLpro enzyme, which possesses deubiquitinating and deISGylating activity (Freitas et al., 2020; Klemm et al., 2020; Freitas et al., 2022). Researchers have pointed out that PLpro’s capacity to disengage ISG15 from ISGylated modifications may prove to be critical to the virus’s replicative cycle (Swaim et al., 2021; Große et al., 2022). Moreover, PLpro’s de-ISGylation activity impinges significantly on immune responses. On the one hand, the process of de-ISGylation by PLpro in macrophages, coupled with an escalation of extracellular free ISG15, can engender a perturbed macrophage response (Cao, 2021; Munnur et al., 2021). On the other hand, PLpro’s de-ISGylation activity on components of the IFN signaling pathway, such as melanoma differentiation-associated protein 5 (MDA5) and interferon responsive factor 3 (IRF3), critically impairs innate immunity’s capacity to combat viral infections (Shin et al., 2020; Liu et al., 2021). The DUB activity of PLpro can hinder innate immune responses by obstructing the initiation and signal transduction of IFN through deubiquitination in diverse RLRs (a type of PRRs) signaling pathway’s proteins (Ran et al., 2022; Sun et al., 2022).

## Conclusion and perspective

**Figure 1:**
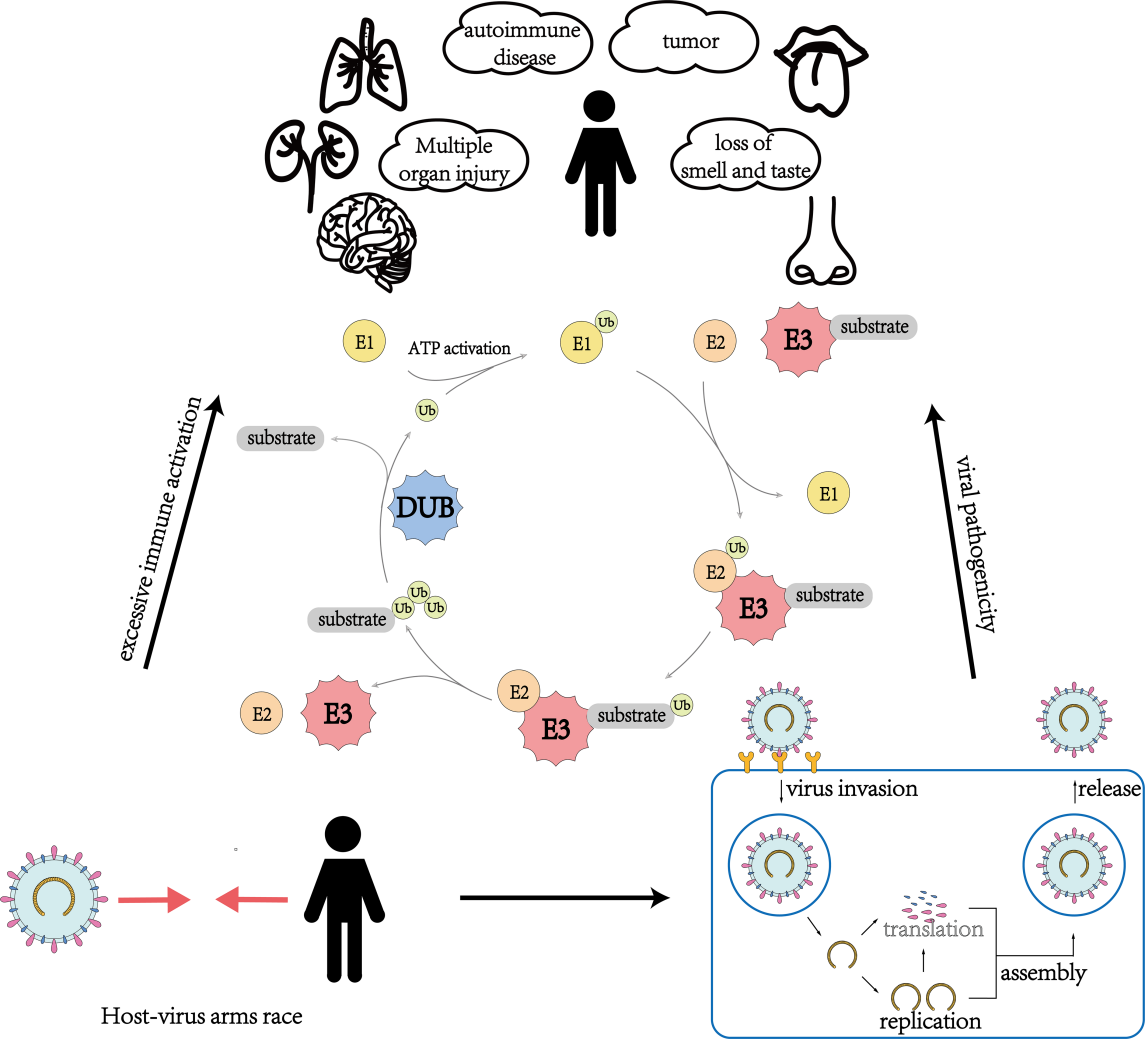
An overview of the role of ubiquitination and deubiquitination involving E3 ubiquitin ligases and deubiquitinating enzymes in multiple aspects of virus-host interactions during the invasion of SARS-CoV-2.

Recent studies have revealed the important role of ubiquitination and deubiquitination processes in the competition between SARS-CoV-2 and the host. The competition for control over key enzymes in the ubiquitin system, such as E3 ligases and deubiquitinases (DUBs), is a critical factor in determining whether a virus can successfully invade its host or whether the host can effectively resist the virus. This review provides a better understanding of the various responses triggered by SARS-CoV-2 infection in the human body from the perspective of ubiquitin modification regulation and insight for potential COVID-19 treatments by targeting critical enzymes such as E3 ligases and DUBs (**Figure 1**; **Table 1**). These approaches aim to weaken the virus’s advantage in competing with the host cells, enhance the host’s resistance to the virus, and prevent excessive immune-inflammatory responses. By intervening in these enzymes, we may be able to limit the virus’s ability to replicate and evade the host immune system, ultimately reducing the severity of the infection. However, it is important to note that this approach is still in its early stages of research and development, and further studies are needed to fully understand its potential therapeutic benefits.

**Table 1:** E3 ligases and DUBs in SARS-CoV-2 infection.

E3 ligases/DUBs	Substrate/Interactor	Biological process	Reference
DCAF1	TMPRSS2	virus invasion	(Chen et al., 2021)
Skp2	ACE2	virus invasion	(Wang et al., 2021; Xiao et al., 2023)
UBR4	ACE2	virus invasion	(Chuang et al., 2022)
PIAS4	ACE2	virus invasion	(Jin et al., 2022)
ZNF598	nsp12	virus replication	(Maimaitiyiming et al., 2022)
RNF20	SREBP1	virus replication	(Zhang et al., 2021)
RNF5	virus M&E protein	virus assembly and release	(Yuan et al., 2021; Li et al., 2023)
MARCH8	virus S protein	Host antiviral response	(Lun et al., 2021; Yanzhao et al., 2022)
TRIM21	virus S protein	Host antiviral response	(Chatterjee et al., 2020)
TRIM7	nsp5&nsp8	Host antiviral response	(Liang et al., 2022)
Cullin5	multiple substrates	SARS-CoV-2 hijacking	(Israeli et al., 2022)
CHIP	IRF1	IFN response	(Han et al., 2022)
TRIM6	DHX16	IFN response	(Hage et al., 2022)
TRIM25	RIG-I	IFN response resistance	(Wu et al., 2020; Zhao et al., 2021)
STUB1	RIG-I	IFN response resistance	(Zhang et al., 2021)
nsp5	MAVS	IFN response resistance, excessive inflammation	(Liu et al., 2021; Li et al., 2021)
TRIM31	MAVS	IFN response resistance	(Wang et al., 2021)
elongins-B/C	JAK2	IFN response resistance	(Rong et al., 2021)
cIAP1	ZBP1	excessive inflammation	(Peng et al., 2022)
LUBAC	ZBP1	excessive inflammation	(Peng et al., 2022)
TRIM13	nsp6	excessive inflammation	(Nishitsuji et al., 2022)
RNF121	ORF7a	excessive inflammation	(Nishitsuji et al., 2022)
TRIM59	STAT3	excessive inflammation	(Cai et al., 2023)
CRL2	IFT46,ORF10	indirect effect	(Bing et al., 2022; Wang et al., 2022)
UCHL1	ACE2	virus invasion	(Bednash et al., 2023)
POH1	virus M&E protein	virus assembly and release	(Yuan et al., 2021)
USP13	nsp13	SARS-CoV-2 hijacking	(Süt, 2020; Guo et al., 2021)
USP25	nsp16 and its associated complex	SARS-CoV-2 hijacking	(Alshiraihi et al., 2021)
USP29	ORF9b	SARS-CoV-2 hijacking	(Han et al., 2021)
USP22	STING	IFN response	(Karlowitz et al., 2022)
USP33	IRF9	excessive inflammation	(Mishra and Banerjea, 2021)

## Author contributions

The topic of this review was proposed by BZ. MIZ and MEZ performed a thorough literature search. The manuscript was drafted by MIZ, MEZ, and ZY. The final manuscript was reviewed and edited by JH, ZZ, and BZ. All authors contributed to the article and approved the submitted version.

## References

[B1] Alshiraihi I. M. Klein G. L. Brown M. A. (2021). Targeting NSP16 methyltransferase for the broad-spectrum clinical management of coronaviruses: managing the next pandemic. Diseases 9 (1), 12. doi: 10.3390/diseases9010012 33535388 PMC7930934

[B2] Amerik A. Y. Hochstrasser M. (2004). Mechanism and function of deubiquitinating enzymes. Biochim. Biophys. Acta (BBA) - Mol. Cell Res. 1695 (1), 189–207. doi: 10.1016/j.bbamcr.2004.10.003 15571815

[B3] Bagheri S. H. Asghari A. Farhadi M. Shamshiri A. R. Kabir A. Kamrava S. K.. (2020). Coincidence of COVID-19 epidemic and olfactory dysfunction outbreak in Iran. Med. J. Islam Repub Iran 34, 62. doi: 10.34171/mjiri.34.62 32974228 PMC7500422

[B4] Bednash J. S. Johns F. Farkas D. Elhance A. Adair J. Cress K.. (2023). Inhibiting the deubiquitinase UCHL1 reduces SARS-CoV-2 viral uptake by ACE2. Am. J. Respir. Cell Mol. Biol. 68:5, 480–481. doi: 10.1165/rcmb.2022-0331OC 36730646 PMC10174169

[B5] Belouzard S. Millet J. K. Licitra B. N. Whittaker G. R. (2012). Mechanisms of coronavirus cell entry mediated by the viral spike protein. Viruses 4 (6), 1011–1033. doi: 10.3390/v4061011 22816037 PMC3397359

[B6] Bing Z. Li Y. Feng Q. Song L. Dong C. Yan X. (2022). Structural insights into ORF10 recognition by ZYG11B. Biochem. Biophys. Res. Commun. 616, 14–18. doi: 10.1016/j.bbrc.2022.05.069 35636250 PMC9121654

[B7] Cai H. Chen Y. Feng Y. Asadi M. Kaufman L. Lee K.. (2023). SARS-CoV-2 viral protein ORF3A injures renal tubules by interacting with TRIM59 to induce STAT3 activation. Mol. Ther. 31 (3), 774–787. doi: 10.1016/j.ymthe.2022.12.008 36523164 PMC9750503

[B8] Cao X. (2021). ISG15 secretion exacerbates inflammation in SARS-CoV-2 infection. Nat. Immunol. 22 (11), 1360–1362. doi: 10.1038/s41590-021-01056-3 34671145

[B9] Cao Z. Xia H. Rajsbaum R. Xia X. Wang H. Shi P. Y. (2021). Ubiquitination of SARS-CoV-2 ORF7a promotes antagonism of interferon response. Cell Mol. Immunol. 18 (3), 746–748. doi: 10.1038/s41423-020-00603-6 33473190 PMC7815971

[B10] Chatterjee P. Ponnapati M. Kramme C. Plesa A. M. Church G. M. Jacobson J. M. (2020). Targeted intracellular degradation of SARS-CoV-2 *via* computationally optimized peptide fusions. Commun. Biol. 3 (1), 715. doi: 10.1038/s42003-020-01470-7 33230174 PMC7683566

[B11] Chen D.-Y. Khan N. Close B. J. Goel R. K. Blum B. Tavares A. H.. (2021). SARS-CoV-2 disrupts proximal elements in the JAK-STAT pathway. J. Virol. 95 (19), e0086221. doi: 10.1128/JVI.00862-21 34260266 PMC8428404

[B12] Chen Y. Lear T. B. Evankovich J. W. Larsen M. B. Lin B. Alfaras I.. (2021). A high-throughput screen for TMPRSS2 expression identifies FDA-approved compounds that can limit SARS-CoV-2 entry. Nat. Commun. 12 (1), 3907. doi: 10.1038/s41467-021-24156-y 34162861 PMC8222394

[B13] Chuang H.-C. Hsueh C. H. Hsu P. M. Huang R.-H. Tsai C.-Y. Chung N.-H.. (2022). SARS-CoV-2 spike protein enhances MAP4K3/GLK-induced ACE2 stability in COVID-19. EMBO Mol. Med. 14 (9), e15904. doi: 10.15252/emmm.202215904 35894122 PMC9353388

[B14] Corse E. Machamer C. E. (2003). The cytoplasmic tails of infectious bronchitis virus e and m proteins mediate their interaction. Virology 312 (1), 25–34. doi: 10.1016/S0042-6822(03)00175-2 12890618 PMC7127533

[B15] Cui Y. Chen F. Gao J. Lei M. Wang D. Jin X.. (2021). Comprehensive landscape of the renin-angiotensin system in pan-cancer: a potential downstream mediated mechanism of SARS-CoV-2. Int. J. Biol. Sci. 17 (14), 3795–3817. doi: 10.7150/ijbs.53312 34671200 PMC8495399

[B16] Daulny A. Tansey W. P. (2009). Damage control: DNA repair, transcription, and the ubiquitin-proteasome system. DNA Repair (Amst) 8 (4), 444–448. doi: 10.1016/j.dnarep.2009.01.017 19272841

[B17] De Lorenzo R. Loré N. I. Finardi A. Mandelli A. Cirillo D. M. Tresoldi C.. (2021). Blood neurofilament light chain and total tau levels at admission predict death in COVID-19 patients. J. Neurol. 268 (12), 4436–4442. doi: 10.1007/s00415-021-10595-6 33973106 PMC8108733

[B18] Freitas B. T. Ahiadorme D. A. Bagul R. S. Durie I. A. Ghosh S. Hill J.. (2022). Exploring noncovalent protease inhibitors for the treatment of severe acute respiratory syndrome and severe acute respiratory syndrome-like coronaviruses. ACS Infect. Dis. 8 (3), 596–611. doi: 10.1021/acsinfecdis.1c00631 35199517 PMC8887654

[B19] Freitas B. T. Durie I. A. Murray J. Longo J. E. Miller H. C. Crich D.. (2020). Characterization and noncovalent inhibition of the deubiquitinase and deISGylase activity of SARS-CoV-2 papain-like protease. ACS Infect. Dis. 6 (8), 2099–2109. doi: 10.1021/acsinfecdis.0c00168 32428392

[B20] Gao X. Liu Y. Zou S. Liu P. Zhao J. Yang C.. (2021). Genome-wide screening of SARS-CoV-2 infection-related genes based on the blood leukocytes sequencing data set of patients with COVID-19. J. Med. Virol. 93 (9), 5544–5554. doi: 10.1002/jmv.27093 34009691 PMC8242610

[B21] Gao W. Wang L. Ju X. Zhao S. Li Z. Su M.. (2022). The deubiquitinase USP29 promotes SARS-CoV-2 virulence by preventing proteasome degradation of ORF9b. mBio. 13 (3), e0130022. doi: 10.1128/mbio.01300-22 35638730 PMC9239186

[B22] Große M. Setz C. Rauch P. Auth J. Morokutti-Kurz M. Temchura V.. (2022). Inhibitors of deubiquitinating enzymes interfere with the SARS-CoV-2 papain-like protease and block virus replication *In vitro* . Viruses 14 (7), 1404. doi: 10.3390/v14071404 35891385 PMC9324251

[B23] Gülden Baskol Özel M. Saracoglu H. Ulger B. Kalin Unuvar G. Onuk S.. (2022). New avenues to explore in SARS-CoV-2 infection: both TRIM25 and TRIM56 positively correlate with VEGF, GAS6, and sAXL in COVID-19 patients. Viral Immunol. 35 (10), 690–699. doi: 10.1089/vim.2022.0112 36450108

[B24] Guo G. Gao M. Gao X. Zhu B. Huang J. Luo K.. (2021). SARS-CoV-2 non-structural protein 13 (nsp13) hijacks host deubiquitinase USP13 and counteracts host antiviral immune response. Signal Transduct Target Ther. 6 (1), 119. doi: 10.1038/s41392-021-00509-3 33707416 PMC7947159

[B25] Hage A. Bharaj P. van Tol S. Giraldo M. I. Gonzalez-Orozco M. Valerdi K. M.. (2022). The RNA helicase DHX16 recognizes specific viral RNA to trigger RIG-i-dependent innate antiviral immunity. Cell Rep. 38 (10), 110434. doi: 10.1016/j.celrep.2022.110434 35263596 PMC8903195

[B26] Han Y Bai X. Liu S. Zhu J. Zhang F. Xie L.. (2022). XAF1 protects host against emerging RNA viruses by stabilizing IRF1-dependent antiviral immunity. J. Virol. 96 (17), e0077422. doi: 10.1128/jvi.00774-22 35972291 PMC9472610

[B27] Han L. Zhuang M. W. Deng J. Zheng Y. Zhang J. Nan M.-L.. (2021). SARS-CoV-2 ORF9b antagonizes type I and III interferons by targeting multiple components of the RIG-I/MDA-5-MAVS, TLR3-TRIF, and cGAS-STING signaling pathways. J. Med. Virol. 93 (9), 5376–5389. doi: 10.1002/jmv.27050 33913550 PMC8242602

[B28] Hoffmann M. Kleine-Weber H. Schroeder S. Krüger N. Herrler T. Erichsen S.. (2020). SARS-CoV-2 cell entry depends on ACE2 and TMPRSS2 and is blocked by a clinically proven protease inhibitor. Cell. 181 (2), 271–280.e8. doi: 10.1016/j.cell.2020.02.052 32142651 PMC7102627

[B29] Huang J. Li R. Cheng J. Zhen P. (2022). Transcriptome analysis of peripheral blood mononuclear cells response in patients with severe COVID-19 reveals crucial genes regulating protein ubiquitination. Med. Sci. Monit. 28, e937532. doi: 10.12659/MSM.937532 36031751 PMC9438463

[B30] Isaacson M. K. Ploegh H. L. (2009). Ubiquitination, ubiquitin-like modifiers, and deubiquitination in viral infection. Cell Host Microbe 5 (6), 559–570. doi: 10.1016/j.chom.2009.05.012 19527883 PMC7103382

[B31] Israeli M. Finkel Y. Yahalom-Ronen Y. Paran N. Chitlaru T. Israeli O.. (2022). Genome-wide CRISPR screens identify GATA6 as a proviral host factor for SARS-CoV-2 *via* modulation of ACE2. Nat. Commun. 13 (1), 2237. doi: 10.1038/s41467-022-29896-z 35469023 PMC9039069

[B32] Jin S. He X. Ma L. Zhuang Z. Wang Y. Lin M.. (2022). Suppression of ACE2 SUMOylation protects against SARS-CoV-2 infection through TOLLIP-mediated selective autophagy. Nat. Commun. 13 (1), 5204. doi: 10.1038/s41467-022-32957-y 36057605 PMC9440653

[B33] Jung J. Baek J. Tae K. Shin D. Han S. Yang W.. (2022). Structural mechanism for regulation of Rab7 by site-specific monoubiquitination. Int. J. Biol. Macromol 194, 347–357. doi: 10.1016/j.ijbiomac.2021.11.074 34801583

[B34] Karlowitz R. Stanifer M. L. Roedig J. Andrieux G. Bojkova D. Bechtel M.. (2022). USP22 controls type III interferon signaling and SARS-CoV-2 infection through activation of STING. Cell Death Dis. 13 (8), 684. doi: 10.1038/s41419-022-05124-w 35933402 PMC9357023

[B35] Kim D. K. Weller B. Lin C. W. Sheykhkarimli D. Knapp J. J. Dugied G.. (2023). A proteome-scale map of the SARS-CoV-2-human contactome. Nat. Biotechnol. 41 (1), 140–149. doi: 10.1038/s41587-022-01475-z 36217029 PMC9849141

[B36] Klemm T. Ebert G. Calleja D. J. Allison C. C. Richardson L. W. Bernardini J. P.. (2020). Mechanism and inhibition of the papain-like protease, PLpro, of SARS-CoV-2. EMBO J. 39 (18), e106275. doi: 10.15252/embj.2020106275 32845033 PMC7461020

[B37] Lee S. Lee Y. S. Choi Y. Son A. Park Y. Lee K.-M.. (2021). The SARS-CoV-2 RNA interactome. Mol. Cell. 81 (13), 2838–2850.e6. doi: 10.1016/j.molcel.2021.04.022 33989516 PMC8075806

[B38] Li M. Ferretti M. Ying B. Descamps H. Lee E. Dittmar M.. (2021). Pharmacological activation of STING blocks SARS-CoV-2 infection. Sci. Immunol. 6 (59), eabi9007. doi: 10.1126/sciimmunol.abi9007 34010142 PMC10021026

[B39] Li Z. Hao P. Zhao Z. Gao W. Huan C. Li L.. (2023). The E3 ligase RNF5 restricts SARS-CoV-2 replication by targeting its envelope protein for degradation. Signal Transduct Target Ther. 8 (1), 53. doi: 10.1038/s41392-023-01335-5 36737599 PMC9897159

[B40] Li W. Qiao J. You Q. Zong S. Peng Q. Liu Y.. (2021). SARS-CoV-2 Nsp5 activates NF-κB pathway by upregulating SUMOylation of MAVS. Front. Immunol. 12, 750969. doi: 10.3389/fimmu.2021.750969 34858407 PMC8631293

[B41] Liang X. Xiao J. Li X. Liu Y. Lu Y. Wen Y.. (2022). A c-terminal glutamine recognition mechanism revealed by E3 ligase TRIM7 structures. Nat. Chem. Biol. 18 (11), 1214–1223. doi: 10.1038/s41589-022-01128-x 35982226

[B42] Liu Z. Fu Y. Huang Y. Zeng F. Rao J. Xiao X.. (2022). Ubiquitination of SARS-CoV-2 ORF7a prevents cell death induced by recruiting BclXL to activate ER stress. Microbiol. Spectr 10 (6), e0150922. doi: 10.1128/spectrum.01509-22 36326498 PMC9769937

[B43] Liu G. Lee J. H. Parker Z. M. Acharya D. Chiang J. J. van Gent M.. (2021). ISG15-dependent activation of the sensor MDA5 is antagonized by the SARS-CoV-2 papain-like protease to evade host innate immunity. Nat. Microbiol. 6 (4), 467–478. doi: 10.1038/s41564-021-00884-1 33727702 PMC8103894

[B44] Liu Y. Qin C. Rao Y. Ngo C. Feng J. J. Zhao J.. (2021). SARS-CoV-2 Nsp5 demonstrates two distinct mechanisms targeting RIG-I and MAVS to evade the innate immune response. mBio. 12 (5), e0233521. doi: 10.1128/mBio.02335-21 34544279 PMC8546575

[B45] Liu Y. Wu Y. Liu B. Zhang Y. San D. Chen Y.. (2021b). Biomarkers and immune repertoire metrics identified by peripheral blood transcriptomic sequencing reveal the pathogenesis of COVID-19. Front. Immunol. 12. doi: 10.3389/fimmu.2021.677025 PMC8421539 34504487

[B46] Lun C. M. Waheed A. A. Majadly A. Powell N. Freed E. O. (2021). Mechanism of viral glycoprotein targeting by membrane-associated RING-CH proteins. mBio. 12 (2), e00219–21. doi: 10.1128/mBio.00219-21 33727347 PMC8092221

[B47] Maimaitiyiming Y. Yang T. Wang Q. Q. Feng Y. Chen Z. Björklund M.. (2022). Heat treatment promotes ubiquitin-mediated proteolysis of SARS-CoV-2 RNA polymerase and decreases viral load. Res. (Wash D C) 2022, 9802969. doi: 10.34133/2022/9802969 PMC8918953 35321260

[B48] Makaronidis J. Mok J. Balogun N. Magee C. G. Omar R. Z. Carnemolla A.. (2020). Seroprevalence of SARS-CoV-2 antibodies in people with an acute loss in their sense of smell and/or taste in a community-based population in London, UK: an observational cohort study. PLoS Med. 17 (10), e1003358. doi: 10.1371/journal.pmed.1003358 33001967 PMC7529306

[B49] McBride R. Van Zyl M. Fielding B. C. (2014). The coronavirus nucleocapsid is a multifunctional protein. Viruses 6 (8), 2991–3018. doi: 10.3390/v6082991 25105276 PMC4147684

[B50] Mishra R. Banerjea A. C. (2021). SARS-CoV-2 spike targets USP33-IRF9 axis *via* exosomal miR-148a to activate human microglia. Front. Immunol. 12, 656700. doi: 10.3389/fimmu.2021.656700 33936086 PMC8079643

[B51] Munnur D. Teo Q. Eggermont D. Lee H. H.Y. Thery F. Ho J.. (2021). Altered ISGylation drives aberrant macrophage-dependent immune responses during SARS-CoV-2 infection. Nat. Immunol. 22 (11), 1416–1427. doi: 10.1038/s41590-021-01035-8 34663977

[B52] Nishitsuji H. Iwahori S. Ohmori M. Shimotohno K. Murata T. (2022). Ubiquitination of SARS-CoV-2 NSP6 and ORF7a facilitates NF-κB activation. mBio 13 (4), e0097122. doi: 10.1128/mbio.00971-22 35856559 PMC9426613

[B53] Peng R. Wang C. K. Wang-Kan X. Idorn M. Kjaer M. Zhou F. Y.. (2022). Human ZBP1 induces cell death-independent inflammatory signaling *via* RIPK3 and RIPK1. EMBO Rep. 23 (12), e55839. doi: 10.15252/embr.202255839 36268590 PMC9724671

[B54] Perng Y. C. Lenschow D. J. (2018). ISG15 in antiviral immunity and beyond. Nat. Rev. Microbiol. 16 (7), 423–439. doi: 10.1038/s41579-018-0020-5 29769653 PMC7097117

[B55] Prescott L. (2022). SARS-CoV-2 3CLpro whole human proteome cleavage prediction and enrichment/depletion analysis. Comput. Biol. Chem. 98, 107671. doi: 10.1016/j.compbiolchem.2022.107671 35429835 PMC8958254

[B56] Ran X.-H. Zhu J. W. Chen Y. Y. Ni R. Z. Mu D. (2022). Papain-like protease of SARS-CoV-2 inhibits RLR signaling in a deubiquitination-dependent and deubiquitination-independent manner. Front. Immunol. 13, 947272. doi: 10.3389/fimmu.2022.947272 36032116 PMC9411789

[B57] Ravindran V. Wagoner J. Athanasiadis P. Den Hartigh A. B. Sidorova J. M. Ianevski A.. (2022). Discovery of host-directed modulators of virus infection by probing the SARS-CoV-2-host protein-protein interaction network. Brief Bioinform. 23 (6), bbac456. doi: 10.1093/bib/bbac456 36305426 PMC9677461

[B58] Reed S. I. (2006). The ubiquitin-proteasome pathway in cell cycle control. Results Probl Cell Differ. 42, 147–181. doi: 10.1007/b136681 16903211

[B59] Rong W. Yang X. Chang M. Xue Z. Wang W. Bai L.. (2021). ORF3a protein of severe acute respiratory syndrome coronavirus 2 inhibits interferon-activated janus Kinase/Signal transducer and activator of transcription signaling *via* elevating suppressor of cytokine signaling 1. Front. Microbiol. 12, 752597. doi: 10.3389/fmicb.2021.752597 34650546 PMC8506155

[B60] Rothwarf D. M. Zandi E. Natoli G. Karin M. (1998). IKK-gamma is an essential regulatory subunit of the IkappaB kinase complex. Nature 395 (6699), 297–300. doi: 10.1038/26261 9751060

[B61] Rui Y. Su J. Shen S. Hu Y. Huang D. Zheng W.. (2021). Unique and complementary suppression of cGAS-STING and RNA sensing- triggered innate immune responses by SARS-CoV-2 proteins. Signal Transduct Target Ther. 6 (1), 123. doi: 10.1038/s41392-021-00515-5 33723219 PMC7958565

[B62] Schoeman D. Fielding B. C. (2019). Coronavirus envelope protein: current knowledge. Virol. J. 16 (1), 69. doi: 10.1186/s12985-019-1182-0 31133031 PMC6537279

[B63] Schwartzenburg J. Reed R. Koul H. Zea A. H. Shellito J. Miele L.. (2022). ISGylation is increased in the peripheral blood mononuclear cells derived from symptomatic COVID-19 patients. Exp. Biol. Med. (Maywood) 247 (10), 842–847. doi: 10.1177/15353702221075606 35130743 PMC9160937

[B64] Selvaraj G. Kaliamurthi S. Peslherbe G. H. Wei D. Q. (2021). Identifying potential drug targets and candidate drugs for COVID-19: biological networks and structural modeling approaches. F1000Res 10, 127. doi: 10.12688/f1000research.50850.3 33968364 PMC8080978

[B65] Serrano-Maciá M. Lachiondo-Ortega S. Iruzubieta P. Goikoetxea-Usandizaga N. Bosch A. Egia-Mendikute L.. (2022). Neddylation tunes peripheral blood mononuclear cells immune response in COVID-19 patients. Cell Death Discovery 8 (1), 316. doi: 10.1038/s41420-022-01115-0 35831294 PMC9277603

[B66] Seth R. B. Sun L. Ea C. K. Chen Z. J. (2005). Identification and characterization of MAVS, a mitochondrial antiviral signaling protein that activates NF-kappaB and IRF 3. Cell 122 (5), 669–682. doi: 10.1016/j.cell.2005.08.012 16125763

[B67] Shin D. Mukherjee R. Grewe D. Bojkova D. Baek K. Bhattacharya A.. (2020). Papain-like protease regulates SARS-CoV-2 viral spread and innate immunity. Nature 587 (7835), 657–662. doi: 10.1038/s41586-020-2601-5 32726803 PMC7116779

[B68] Somasekharan S. P. Gleave M. (2021). SARS-CoV-2 nucleocapsid protein interacts with immunoregulators and stress granules and phase separates to form liquid droplets. FEBS Lett. 595 (23), 2872–2896. doi: 10.1002/1873-3468.14229 34780058 PMC8652540

[B69] Song P. Li W. Xie J. Hou Y. You C. (2020). Cytokine storm induced by SARS-CoV-2. Clin. Chim. Acta 509, 280–287. doi: 10.1016/j.cca.2020.06.017 32531256 PMC7283076

[B70] Storici F. (2011). “The Ubiquitin-Proteasome System and DNA Repair,” in DNA Repair: on the pathways to fixing DNA damage and errors. BoD – Books Demand, 255–286.

[B71] Stukalov A. Girault V. Grass V. Karayel O. Bergant V. Urban C.. (2021). Multilevel proteomics reveals host perturbations by SARS-CoV-2 and SARS-CoV. Nature 594 (7862), 246–252. doi: 10.1038/s41586-021-03493-4 33845483

[B72] Sui L. Zhao Y. Wang W. Wu P. Wang Z. Yu Y.. (2021). SARS-CoV-2 membrane protein inhibits type I interferon production through ubiquitin-mediated degradation of TBK1. Front. Immunol. 12, 662989. doi: 10.3389/fimmu.2021.662989 34084167 PMC8168463

[B73] Sun T. Liu Z. Yang Q. (2020). The role of ubiquitination and deubiquitination in cancer metabolism. Mol. Cancer 19 (1), 146. doi: 10.1186/s12943-020-01262-x 33004065 PMC7529510

[B74] Sun X. Quan L. Chen R. Liu D. (2022). Direct interaction of coronavirus nonstructural protein 3 with melanoma differentiation-associated gene 5 modulates type I interferon response during coronavirus infection. Int. J. Mol. Sci. 23 (19), 11692. doi: 10.3390/ijms231911692 36232993 PMC9570369

[B75] Süt B. B. (2020). Molecular profiling of immune cell-enriched severe acute respiratory syndrome coronavirus 2 (SARS-CoV-2) interacting protein USP13. Life Sci. 258, 118170. doi: 10.1016/j.lfs.2020.118170 32735883 PMC7387267

[B76] Swaim C. D. Dwivedi V. Perng Y. C. Zhao X. Canadeo L. A. Harastani H. H.. (2021). 6-thioguanine blocks SARS-CoV-2 replication by inhibition of PLpro. iScience 24 (10), 103213. doi: 10.1016/j.isci.2021.103213 34632326 PMC8487320

[B77] Temena M. A. Acar A. (2022). Increased TRIM31 gene expression is positively correlated with SARS-CoV-2 associated genes TMPRSS2 and TMPRSS4 in gastrointestinal cancers. Sci. Rep. 12 (1), 11763. doi: 10.1038/s41598-022-15911-2 35970857 PMC9378649

[B78] Tokic D. Mikacic M. Kumric M. Ticinovic Kurir T. Rancic I. Martinovic D.. (2022). Association between brain injury markers and testosterone in critically-ill COVID-19 Male patients. Microorganisms 10 (11), 2095. doi: 10.3390/microorganisms10112095 36363686 PMC9697553

[B79] Vanderboom P. M. Mun D. G. Madugundu A. K. Mangalaparthi K. K. Saraswat M. Garapati K.. (2021). Proteomic signature of host response to SARS-CoV-2 infection in the nasopharynx. Mol. Cell Proteomics 20, 100134. doi: 10.1016/j.mcpro.2021.100134 34400346 PMC8363427

[B80] Vetter P. Vu D. L. L’Huillier A. G. Schibler M. Kaiser L. Jacquerioz F. (2020). Clinical features of covid-19. BMJ 369, m1470. doi: 10.1136/bmj.m1470 32303495

[B81] Wang S. Dai T. Qin Z. Pan T. Chu F. Lou L.. (2021). Targeting liquid-liquid phase separation of SARS-CoV-2 nucleocapsid protein promotes innate antiviral immunity by elevating MAVS activity. Nat. Cell Biol. 23 (7), 718–732. doi: 10.1038/s41556-021-00710-0 34239064

[B82] Wang L. Liu C. Yang B. Zhang H. Jiao J. Zhang R.. (2022). SARS-CoV-2 ORF10 impairs cilia by enhancing CUL2ZYG11B activity. J. Cell Biol. 221 (7), e202108015. doi: 10.1083/jcb.202108015 35674692 PMC9184850

[B83] Wang Q. Wu J. Wang H. Gao Y. Liu Q. Mu A.. (2020). Structural basis for RNA replication by the SARS-CoV-2 polymerase. Cell. 182 (2), 417–428.e13. doi: 10.1016/j.cell.2020.05.034 32526208 PMC7242921

[B84] Wang J. Y. Zhang W. Roehrl M. W. Roehrl V. B. Roehrl M. H. (2021). An autoantigen profile of human A549 lung cells reveals viral and host etiologic molecular attributes of autoimmunity in COVID-19. J. Autoimmun. 120, 102644. doi: 10.1016/j.jaut.2021.102644 33971585 PMC8075847

[B85] Wang G. Zhao Q. Zhang H. Liang F. Zhang C. Wang J.. (2021). Degradation of SARS-CoV-2 receptor ACE2 by the E3 ubiquitin ligase Skp2 in lung epithelial cells. Front. Med. 15 (2), 252–263. doi: 10.1007/s11684-021-0837-6 33511555 PMC7843238

[B86] WHO COVID-19 Dashboard (2020) (Geneva: World Health Organization). Available at: https://covid19.who.int/.

[B87] Wu Y. Ma L. Zhuang Z. Cai S. Zhao Z. Zhou L.. (2020). Main protease of SARS-CoV-2 serves as a bifunctional molecule in restricting type I interferon antiviral signaling. Signal Transduct Target Ther. 5 (1), 221. doi: 10.1038/s41392-020-00332-2 33024073 PMC7537955

[B88] Wu J. Shi Y. Pan X. Wu S. Hou R. Zhang Y.. (2021). SARS-CoV-2 ORF9b inhibits RIG-I-MAVS antiviral signaling by interrupting K63-linked ubiquitination of NEMO. Cell Rep. 34 (7), 108761. doi: 10.1016/j.celrep.2021.108761 33567255 PMC7857071

[B89] Xiao Y. Yan Y. Chang L. Ji H. Sun H. Song S.. (2023). CDK4/6 inhibitor palbociclib promotes SARS-CoV-2 cell entry by down-regulating SKP2 dependent ACE2 degradation. Antiviral Res. 212, 105558. doi: 10.1016/j.antiviral.2023.105558 36806814 PMC9938000

[B90] Yadav D. Lee J. Y. Puranik N. Chauhan P. S. Chavda V. Jin J.-O.. (2022). Modulating the ubiquitin–proteasome system: a therapeutic strategy for autoimmune diseases. Cells 11 (7), 1093. doi: 10.3390/cells11071093 35406655 PMC8997991

[B91] Yang H. Rao Z. (2021). Structural biology of SARS-CoV-2 and implications for therapeutic development. Nat. Rev. Microbiol. 19 (11), 685–700. doi: 10.1038/s41579-021-00630-8 34535791 PMC8447893

[B92] Yang Y. Wu Y. Meng X. Wang Z. Younis M. Liu Y.. (2022). SARS-CoV-2 membrane protein causes the mitochondrial apoptosis and pulmonary edema *via* targeting BOK. Cell Death Differ. 29 (7), 1395–1408. doi: 10.1038/s41418-022-00928-x 35022571 PMC8752586

[B93] Yanzhao Z. Ozono S. Tada T. Tobiume M. Kameoka M. Kishigami S.. (2022). MARCH8 targets cytoplasmic lysine residues of various viral envelope glycoproteins. Microbiol. Spectr 10 (1), e0061821. doi: 10.1128/spectrum.00618-21 35019698 PMC8754143

[B94] Yuan Z. Hu B. Xiao H. Tan X. Li Y. Tang K.. (2021). The E3 ubiquitin ligase RNF5 facilitates SARS-CoV-2 membrane protein-mediated virion release. mBio 13 (1), e0316821. doi: 10.1128/mbio.03168-21 35100873 PMC8805027

[B95] Zhang Z. Nomura N. Muramoto Y. Ekimoto T. Uemura T. Liu K.. (2022). Structure of SARS-CoV-2 membrane protein essential for virus assembly. Nat. Commun. 13 (1), 4399. doi: 10.1038/s41467-022-32019-3 35931673 PMC9355944

[B96] Zhang S. Wang J. Cheng G. (2021). Protease cleavage of RNF20 facilitates coronavirus replication via stabilization of SREBP1. Proc. Natl. Acad. Sci. U.S.A. 118 (37), e2107108118. doi: 10.1073/pnas.2107108118 34452991 PMC8449311

[B97] Zhang H. Zheng H. Zhu J. Dong Q. Wang J. Fan H.. (2021). Ubiquitin-modified proteome of SARS-CoV-2-Infected host cells reveals insights into virus-host interaction and pathogenesis. J. Proteome Res. 20 (5), 2224–2239. doi: 10.1021/acs.jproteome.0c00758 33666082

[B98] Zhao Y. Sui L. Wu P. Wang W. Wang Z. Yu Y.. (2021). A dual-role of SARS-CoV-2 nucleocapsid protein in regulating innate immune response. Signal Transduct Target Ther. 6 (1), 331. doi: 10.1038/s41392-021-00742-w 34471099 PMC8409078

